# RETRACTION: Diabetic Foot Wound Ulcer Management by Laser Therapy: A Meta‐Analysis

**DOI:** 10.1111/iwj.70187

**Published:** 2025-01-12

## Abstract

**RETRACTION**: LiuH. , Ya‐QingX. , Cai‐FengY. , Jia‐LiH. , and Xian‐YuT. , “Diabetic Foot Wound Ulcer Management by Laser Therapy: A Meta‐Analysis,” International Wound Journal 20, no. 10 (2023): 4208–4216, 10.1111/iwj.14320.37596719 PMC10681457

The above article, published online on 18 August 2023, in Wiley Online Library (http://onlinelibrary.wiley.com/), has been retracted by agreement between the journal Editor in Chief, Professor Keith Harding; and John Wiley & Sons Ltd. Following an investigation by the publisher, all parties have concluded that this article was accepted solely on the basis of a compromised peer review process. The editors have therefore decided to retract the article. The authors disagree with the retraction.

**RETRACTION**: H. Liu , X. Ya‐Qing , Y. Cai‐Feng , H. Jia‐Li , and T. Xian‐Yu , “Diabetic Foot Wound Ulcer Management by Laser Therapy: A Meta‐Analysis,” International Wound Journal 20, no. 10 (2023): 4208–4216, 10.1111/iwj.14320.37596719 PMC10681457

The above article, published online on 18 August 2023, in Wiley Online Library (http://onlinelibrary.wiley.com/), has been retracted by agreement between the journal Editor in Chief, Professor Keith Harding; and John Wiley & Sons Ltd. Following an investigation by the publisher, all parties have concluded that this article was accepted solely on the basis of a compromised peer review process. The editors have therefore decided to retract the article. The authors disagree with the retraction.

